# Effects of NAD+ precursor supplementation on glucose and lipid metabolism in humans: a meta-analysis

**DOI:** 10.1186/s12986-022-00653-9

**Published:** 2022-03-18

**Authors:** Ou Zhong, Jinyuan Wang, Yongpeng Tan, Xiaocan Lei, Zhihan Tang

**Affiliations:** Hengyang Medical School, University of South China, Hengyang, 421001 China

**Keywords:** NAD+, Nicotinic Acid, Nicotinamide, Nicotinamide mononucleotide, Nicotinamide riboside, Meta-analysis

## Abstract

**Background:**

This meta-analysis was performed to investigate the effects of nicotinamide adenine dinucleotide (NAD+) precursor supplementation on glucose and lipid metabolism in human body.

**Methods:**

PubMed, Embase, CENTRAL, Web of Science, Scopus databases were searched to collect clinical studies related to the supplement of NAD+ precursor from inception to February 2021. Then the retrieved documents were screened, the content of the documents that met the requirements was extracted. Meta-analysis and quality evaluation was performed detection were performed using RevMan5.4 software. Stata16 software was used to detect publication bias, Egger and Begg methods were mainly used. The main research terms of NAD+ precursors were Nicotinamide Riboside (NR), Nicotinamide Mononucleotide (NMN), Nicotinic Acid (NA), Nicotinamide (NAM). The changes in the levels of triglyceride (TG), total cholesterol (TC), low-density lipoprotein (LDL), high-density lipoprotein (HDL), and fasting blood glucose were mainly concerned.

**Results:**

A total of 40 articles were included in the meta-analysis, with a sample of 14,750 cases, including 7406 cases in the drug group and 7344 cases in the control group. The results of meta-analysis showed that: NAD+ precursor can significantly reduce TG level (SMD = − 0.35, 95% CI (− 0.52, − 0.18), *P* < 0.0001), and TC (SMD = − 0.33, 95% CI (− 0.51, − 0.14), *P* = 0.0005), and LDL (SMD = − 0.38, 95% CI (− 0.50, − 0.27), *P* < 0.00001), increase HDL level (SMD = 0.66, 95% CI (0.56, 0.76), *P* < 0.00001), and plasma glucose level in the patients (SMD = 0.27, 95% CI (0.12, 0.42), *P* = 0.0004). Subgroup analysis showed that supplementation of NA had the most significant effect on the levels of TG, TC, LDL, HDL and plasma glucose.

**Conclusions:**

In this study, a meta-analysis based on currently published clinical trials with NAD+ precursors showed that supplementation with NAD+ precursors improved TG, TC, LDL, and HDL levels in humans, but resulted in hyperglycemia, compared with placebo or no treatment. Among them, NA has the most significant effect on improving lipid metabolism. In addition, although NR and NAM supplementation had no significant effect on improving human lipid metabolism, the role of NR and NAM could not be directly denied due to the few relevant studies at present. Based on subgroup analysis, we found that the supplement of NAD+ precursors seems to have little effect on healthy people, but it has a significant beneficial effect on patients with cardiovascular disease and dyslipidemia. Due to the limitation of the number and quality of included studies, the above conclusions need to be verified by more high-quality studies.

**Supplementary Information:**

The online version contains supplementary material available at 10.1186/s12986-022-00653-9.

## Background

Nicotinamide adenine dinucleotide (NAD+) is an important cofactor of redox reaction and a central regulator of various metabolisms in the human body. It is involved in a variety of biological processes and a class of substances necessary for energy production, fatty acid and cholesterol synthesis, oxidation reaction, ATP generation, gluconeogenesis and keto generation [1, 2]. There are two major NAD+ synthesis pathways in the human body: de novo synthesis and salvage from precursors. The de novo synthesis of NAD+ converts tryptophan to quinolinic acid (QA) through the kynurenine pathway. The salvage pathways are mainly through the recovery of nicotinamide mononucleotide (NMN), nicotinamide riboside (NR), nicotinamide (NAM), and nicotinic acid (NA). To maintain a certain level of NAD+ in the body, most NAD+ is produced by the salvage pathways, rather than de novo synthesis [3]. Sirtuins are a family of NAD+ -dependent protein deacetylases (SIRT1-7). In 1999, Frye discovered that mammalian sirtuins metabolize NAD+ [4]. Since then, sirtuins have been shown to play a major regulatory role in almost all cellular functions, participating in biological processes such as inflammation, cell growth, energy metabolism, circadian rhythm, neuronal function, aging, cancer, obesity, insulin resistance and stress response [3]. The biological role of NAD+ in humans is largely dependent on the presence of the sirtuins [5]. Recent studies have shown that decreased sirtuin6 (SIRT6) levels and function are associated with abnormal glucose and lipid metabolism [6]. Nicotinic acid reverses cholesterol transport through sirtuins-dependent deacetylation, resulting in the alternating expression of apolipoprotein, transporter, and protein, which affects human lipid metabolism [5]. Previous studies reported that niacinamide intervention had no significant effect on human lipid metabolism or increased triglyceride (TG), total cholesterol (TC), and low-density lipoprotein (LDL) levels [7, 8]. However, in recent years, more and more studies have shown that NAD+ precursor nicotinamide can significantly improve the level of blood lipid in patients [9–11], suggesting a potential prospect for the treatment of hyperlipidemia. NMN also showed similar effects in mouse models, but the clinical studies on NMN intervention are limited at present, and the relationship between NMN and human lipid metabolism is not clear. Therefore, this meta-analysis was based on existing clinical trials to analyze and evaluate the effects of various NAD+ precursors supplementation on human lipid and glucose metabolism.

## Methods

### Search strategy

PubMed, Embase, CENTRAL, Web of Science, Scopus databases were searched to collect clinical studies related to the supplement of NAD+ precursor from inception to February 2021. The search was carried out by combining subject words and free words. See Additional file 1: Appendix for detailed search words.

### Inclusion and exclusion criteria

Inclusion criteria: (1) Study content: clinical trials of NAD+ precursor supplementation; (2) Type of study: randomized controlled trial (RCT); (3) Intervention: NAD+ precursor supplementation, regardless of dose or other background therapy; control: Placebo or no therapy, and background treatment consistent with the intervention group.

Exclusion criteria: (1) Duplicate publications; (2) Animal experiments, cell experiments, reviews, conference abstracts and other literatures without available data; (3) Literatures with poor quality and obvious statistical errors.

### Literature screening, data extraction and risk of bias assessment

The search, data extraction, and quality assessment were completed independently by 2 reviewers according to inclusion and exclusion criteria. The following information was obtained from each trial: (1) Basic information of the included studies: study title, first author, year of publication, study location, etc.; (2) Baseline characteristics of the subjects and intervention measures in the RCT study; (3) Key elements of bias risk assessment; (4) Drugs used in the trial, duration of follow-up, main outcome indicators, etc. The data collection and assessment were performed independently by two investigators, wherein any disagreements were resolved by discussion. The risk of bias was assessed using the Cochrane handbook.

### Statistical analysis

Statistical meta-analyses were performed using the RevMan5.4 software. Confidence intervals (CIs) were set at 95%. Continuous data were calculated with Standardized Mean Difference (SMD), and CIs were set at 95%, *P* < 0.05 was considered statistically significant. SMD for all outcomes was calculated, using the random effect model due to the significant heterogeneity in the included studies. Stata16 software was used to detect publication bias, Egger and Begg methods were mainly used, *P* > 0.05 indicates no significant publication bias (because Egger examination is more sensitive when the two results are contradictory, the Egger examination results are given priority). If the change value before and after the intervention was not given in the paper, the formula ([SD change = √SD before 2 + SD after 2 − (2*R*SD before *SD after)] (R = 0.5) was used to estimate the change value.

## Results

### Study selection

**Fig. 1:**
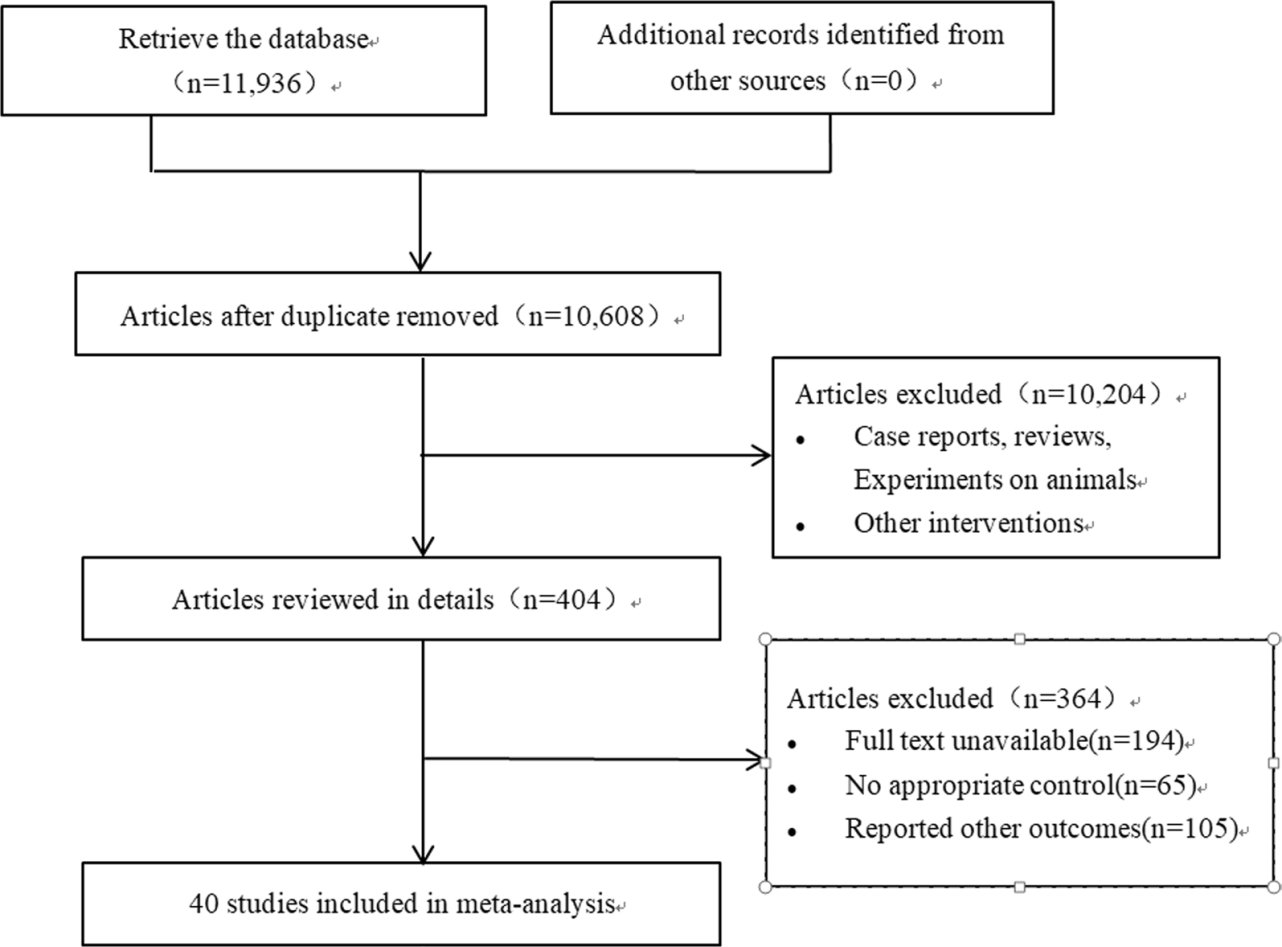
Flowchart of study selection

We identified 11,938 articles in the initial retrieval, including PubMed (n = 1248), Embase (n = 1088), The CENTRAL (n = 4512), Web of Science (n = 3097) and Scopus (n = 1991). Of these, 1328 duplicate articles were excluded after carefully examining the titles and abstracts. After screening, 40 studies were included in the meta-analysis, the literature screening process and results are shown in Fig. 1.

### Study characteristics and quality evaluation

**Fig. 2:**
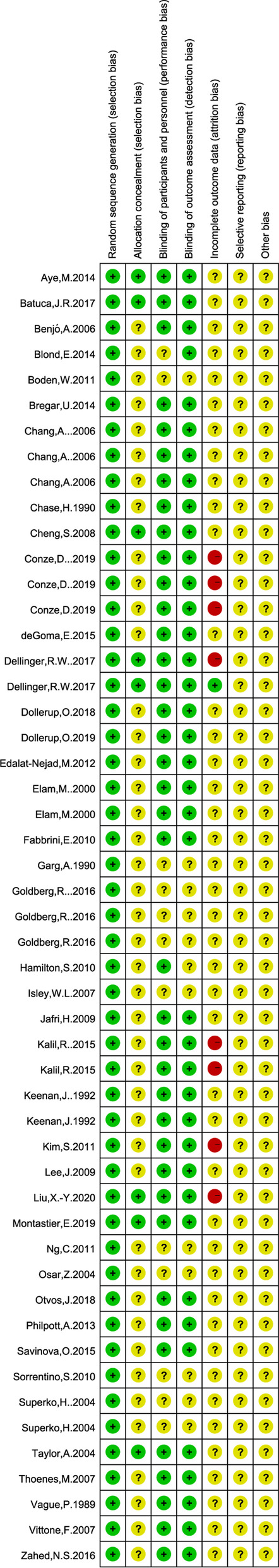
Quality assessment chart

The baseline characteristics of studies and patients are shown in Table 1. A total of 40 articles were included, with a sample of 14,750 cases, including 7406 cases in the drug group and 7344 cases in the control group. In the included studies, there were 35 NA supplements, 3 NR supplements, 2 NAMs, and 0 NMNs. The evaluation results of bias risk are shown in Fig. 2.

**Table 1:** General characteristics of the included studies

Study	Country	Study design	Population size		The basic characteristics Age, year BMI, kg/m^2^	Intervention		Follow-up	Subgroup classification
			T	C		T	C		
Liu, X.-Y. 2020 [9]	China	Parallel double blind	49	49	Age (T/C)55 ± 2/56 ± 2	NAM 500–1500 mg/d	P	52 weeks	(2)
Conze, D. 2019 [1]	USA	Parallel double blind	35	34	Age (T/C)52.3 ± 5.9/50.7 ± 5.6 BMI (T/C)28 ± 2/28 ± 2	NR 100 mg/d	P	56 days	(1)
Conze, D. 2019 [1]	USA	Parallel double blind	35	34	Age (T/C)50.2 ± 5.8/50.7 ± 5.6 BMI (T/C)28 ± 1/28 ± 2	NR 300 mg/d	P	56 days	(1)
Conze, D/ 2019 [1]	USA	Parallel double blind	35	34	Age (T/C)50.9 ± 5.6/50.7 ± 5.6 BMI (T/C)28 ± 2/28 ± 2	NR 1000 mg/d	P	56 days	(1)
Dollerup, O. 2019 [21]	Denmark	Parallel double blind	20	20	Age (T/C)58 ± 1.6/60 ± 2.0 BMI (T/C)32.4 ± 0.5/33.3 ± 0.6	NR 2000 mg/d	P	12 weeks	(6)
Montastier, E. 2019 [23]	France	Parallel double blind	11	11	Patients are sedentary obese men Age (T/C)35.4 ± 2.2/35.4 ± 1.5 BMI (T/C)33.3 ± 0.7/32.6 ± 0.7	ERN 2000 mg/d	P	8 weeks	(6)
Dollerup, O. 2018 [24]	Denmark	Parallel double blind	20	20	Age (T/C)58 ± 1.6/60 ± 2.0 BMI (T/C)32.4 ± 0.5/33.3 ± 0.6	NR 2000 mg/d	P	12 weeks	(1)
Otvos, J. 2018 [25]	USA	Parallel double blind	1367	1387	Age (T/C)63.5 ± 8.8/63.8 ± 8.7	Statin + ERN	P + Statin	1 year	(5)
Dellinger, R. W. 2017 [8]	Canada	Parallel double blind	40	40	Age 60–80 BMI 18–35	NR 250 mg/d + PT 50 mg/d	P	60 days	(1)
Dellinger, R. W. 2017 [8]	Canada	Parallel double blind	40	40	Age 60–80 BMI 18–35	NR 500 mg/d + PT 100 mg/d	P	60 days	(1)
Batuca, J. R. 2017 [26]	Portugal	Parallel double blind	8	9	Age (T/C)46.13 ± 12.02/52.44 ± 9.55 BMI (T/C) 28.09 ± 4.68/29.09 ± 3.2	ERN 1500 mg/d	P	12 weeks	(3)
Goldberg, R. 2016 [27]	US Canada	Parallel	423	410	Patients with normal fasting glucose Age 62.9 ± 9.2 BMI 29.8 ± 5.0	ERN 2000 mg/d + simvastatin 40 mg/d	P	1 year	(1)
Goldberg, R. 2016 [27]	US Canada	Parallel	388	415	Patients with impaired fasting glucose Age 63.2 ± 8.7 BMI 31.0 ± 4.8	ERN 2000 mg/d + simvastatin 40 mg/d	P	1 year	(4)
Goldberg, R. 2016 [27]	US Canada	Parallel	547	506	Patients with diabetes Age 64.7 ± 8.3 BMI 32.6 ± 5.7	ERN 2000 mg/d + simvastatin 40 mg/d	P	1 year	(4)
Zahed, N. S. 2016 [28]	Iran	Parallel double blind	35	35	Age (T/C) 49.8 ± 14.6/51.1 ± 14.1	NA 100 mg/d	P	8 weeks	(2)
Savinova, O. 2015 [29]	USA	Parallel double blind	14	14	Patients with the Metabolic Syndrome Age (T/C)47.0 ± 11.3/49.6 ± 12.9 BMI (T/C)32.7 ± 4.6/29.8 ± 2.5	ERN 2000 mg/d	P	16 weeks	(6)
Kalil, R. 2015 [30]	USA	Parallel double blind	254	251	Patients with chronic kidney disease Age (T/C)70.6 ± 7.2/70.8 ± 7.4 BMI (T/C)30.9 ± 5.4/30.4 ± 5.8	ERN 2000 mg/d + Simvastatin 40 mg/d	P + Simvastatin 40 mg/d	1 year	(2)
Kalil, R. 2015 [30]	USA	Parallel double blind	1464	1444	Patients without chronic kidney disease Age 62.5 ± 8.4	ERN 2000 mg/d + Simvastatin 40 mg/d	P + Simvastatin 40 mg/d	1 year	(3)
deGoma, E. 2015 [31]	USA	Parallel double blind	5	3	Patients with coronary artery disease Age 55	niacin 6000 mg/d	P	12 weeks	(5)
Bregar, U. 2014 [32]	The Republic of Slovenia	Parallel double blind	33	30	Patients with coronary heart disease at least 6 months after myocardial infarction Mean age 52.5 years	niacin/laropiprant (1000/20 mg/d for 4 weeks and 2000/40 mg/d there after) All patients were treated with statins	P	12 weeks	(5)
Blond, E. 2014 [33]	France	cross-over Single blind	20	20	Age 46 ± 13 BMI (T/C)31.2 ± 2.2/31.1 ± 2.2	ERN 2 000 mg/d	P	8 weeks	(3)
Aye, M. 2014 [34]	UK	Parallel double blind	13	12	Patients with Polycystic ovary syndrome Age (T/C)31.0 ± 6.33/31.7 ± 6.51 BMI (T/C)35.8 ± 5.55/34.8 ± 5.03	niacin 1000 mg/d + laropiprant 20 mg/d	P	12 weeks	(6)
Philpott, A. 2013 [35]	Canada	Cross-over double blind	66	66	Patients with coronary heart disease Age 58 ± 8.5 BMI 29.9 ± 4.4	ERN 1500 mg/d + atorvastatin 80 mg/d	P + atorvastatin 80 mg/d	3 months	(5)
Edalat-Nejad, M. 2012 [36]	Iran	cross-over double blind	37	37	Age 57 ± 11 years	Niacin 1000 mg/d	P	8 weeks	(2)
Ng, C. 2011 [37]	China	Parallel	80	80	Age (T/C) 58.34 ± 7.12/57.84 ± 8.48	Niacin 1500 mg/d	P	12 weeks	(3)
Kim, S. 2011 [38]	Korea	Parallel double blind	25	22	Age (T/C) 57.4 ± 6.8/61.8 ± 8.3	ERN 500 mg/d for first 4 weeks and ERN 1000 mg/d for the next 4 weeks	P	8 weeks	(3)
Boden, W. 2011 [39]	USA Canada	Parallel	1561	1554	Age (T/C) 63.7 ± 8.8/63.7 ± 8.7	ERN 1500–2000 mg/d + Simvastatin 40–80 mg/d + Ezetimibe 10 mg/d	P + Simvastatin 40–80 mg/d + Ezetimibe 10 mg/d	1 year	(6)
Fabbrini, E. 2010 [40]	USA	Parallel double blind	9	9	Age (T/C) 43 ± 5/45 ± 3 BMI (T/C)35.8 ± 1.4/37.2 ± 2.0	ERN 2000 mg/d	P	16 weeks	(6)
Sorrentino, S. 2010 [41]	Switzerland	Parallel double blind	15	15	Age (T/C) 58 ± 11/62 ± 9 BMI (T/C)32 ± 4/34 ± 5	ERN 1500 mg/d	P	3 months	(6)
Hamilton, S. 2010 [42]	Australia	Parallel double blind	7	8	Age 65 ± 7 BMI 30 ± 5	Niacin 1500 mg/d	no therapy	20 weeks	(4)
Lee, J. 2009 [43]	UK	Parallel double blind	22	29	Age (T/C) 65 ± 9/65 ± 9 BMI (T/C)31 ± 5/30 ± 5	NA 1000 mg/d for first 4 weeks, 1500 mg/d for a further 4 weeks, and then 2000 mg/d for the remainder	P	12 months	(6)
Jafri, H. 2009 [44]	USA	Parallel double blind	27	27	Age (T/C) 60 ± 10/57 ± 7	ERN 1000 mg/d	P	3 months	(5)
Cheng, S. 2008 [10]	USA	Cross-over double blind	33	33	Hemodialysis patients with phosphorus levels > 5.0 mg/dl Age (T/C) 52.6/52.6	NAM 1500 mg/d	P	8 weeks	(2)
Vittone, F. 2007 [45]	USA	Parallel double blind	80	80	Age (T/C) 54.0 ± 8/53.4 ± 8 BMI (T/C) 29.7 ± 5/29.4 ± 4	Niacin + simvastatin	P	3 years	(5)
Thoenes, M. 2007 [46]	Germany	Parallel double blind	30	15	Patients with the metabolic syndrome Age (T/C) 34.6 ± 8.1/37.5 ± 9.6 BMI (T/C) 29.7 ± 5/29.4 ± 4	ERN 1000 mg/d	P	52 weeks	(3)
Isley, W. L. 2007 [47]	USA	Parallel	7	7	Age (T/C) 48 ± 14/58 ± 10 BMI (T/C) 31.7 ± 1.5/30.3 ± 2.1	Niacin 3000 mg/d	P	12 weeks	(5)
Chang, A. 2006 [48]	USA	Cross-over double blind	15	15	Patients with normal glucose tolerance Age 26 ± 6 BMI 25 ± 3	NA 2000 mg/d	P	2 weeks	(1)
Chang, A. 2006 [48]	USA	Cross-over double blind	16	16	Patients with normal glucose tolerance Age 70 ± 6 BMI 26 ± 3	NA 2000 mg/d	P	2 weeks	(1)
Chang, A. 2006 [48]	USA	Cross-over double blind	14	14	Patients with impaired glucose tolerance Age 70 ± 6 BMI 25 ± 3	NA 2000 mg/d	P	2 weeks	(4)
Benjó, A. 2006 [49]	Brazil	Parallel double blind	11	11	Patients with low HDL-cholesterol BMI (T/C) 27.4 ± 3.7/26.5 ± 3.7	no-flush niacin 1500 mg/d	P	3 months	(1)
Taylor, A. 2004 [50]	USA	Parallel double blind	78	71	Age (T/C) 67 ± 10/68 ± 10	ERN 1000 mg/d	P	12 months	(6)
Osar, Z. 2004 [51]	Turkey	Parallel	15	15	Age (T/C) 55 ± 10/59 ± 8 BMI (T/C) 30 ± 5/28 ± 3	NAM 50 mg/kg	P	1 month	(4)
Superko, H. 2004 [52]	USA	Parallel	60	61	Age (T/C) 53 ± 12/55 ± 12 BMI (T/C) 29 ± 4.4/27 ± 3.6	ERN 1500 mg/d	P	14 weeks	(3)
Superko, H. . 2004 [52]	USA	Parallel	59	61	Age (T/C) 53 ± 11/55 ± 12 BMI (T/C) 28 ± 5.2/27 ± 3.6	IRN 3000 mg/d	P	14 weeks	(3)
Elam, M. 2000 [53]	USA	Parallel double blind	49	50	Patients with diabetes Age 67 ± 7 BMI 28 ± 5	Niacin 3000 mg/d or maximum tolerated dosage	P	18 weeks	(4)
Elam, M. 2000 [53]	USA	Parallel double blind	145	150	Patients without diabetes Age 65 ± 9 BMI 27 ± 5	Niacin 3000 mg/d or maximum tolerated dosage	P	18 weeks	(1)
Keenan, J. 1992 [54]	USA	Parallel double blind	21	26	Age (Mean) 58.7	NA 2000–1500 mg/d	P	24 weeks	(3)
Keenan, J. . 1992 [54]	USA	Parallel double blind	26	12	Age (Mean) 39.9	NA 2000–1500 mg/d	P	24 weeks	(3)
Garg, A. 1990 [55]	USA	Cross-over	13	13	Age 59 ± 1 BMI 29.9 ± 0.7	NA 4500 mg/d	no therapy	8 weeks	(4)
Chase, H. 1990 [56]	USA	Parallel double blind	18	17	Age (T/C) 12.5 ± 3.7/10.8 ± 3.5	slow release NAM (100 mg.age (years)^−1^.day^−1^ up to a maximum of 1.5 g/day)	P	12 months	(4)
Vague, P. 1989 [57]	France	Parallel double blind	11	12	Age (T/C) 29.8 ± 7.3/26.8 ± 6.2	NAM 3000 mg/d	P	9 months	(4)

ERN: extended-release nicotinic acid; IRN: immediate-release niacin; P: Placebo; NRPT: Nicotinamide riboside + pterostilbene; P-OM3: Prescription omega-3 acid ethyl esters; ω-3 FA: ω-3 fatty acids; -: Not reported;

(1) Healthy people; (2) Chronic kidney disease (CKD); (3) Dyslipidemia; (4) Pathoglycemia; (5) Cardiovascular disease; (6) Other

### Effect of NAD+ precursor supplementation on TG level

**Fig. 3:**
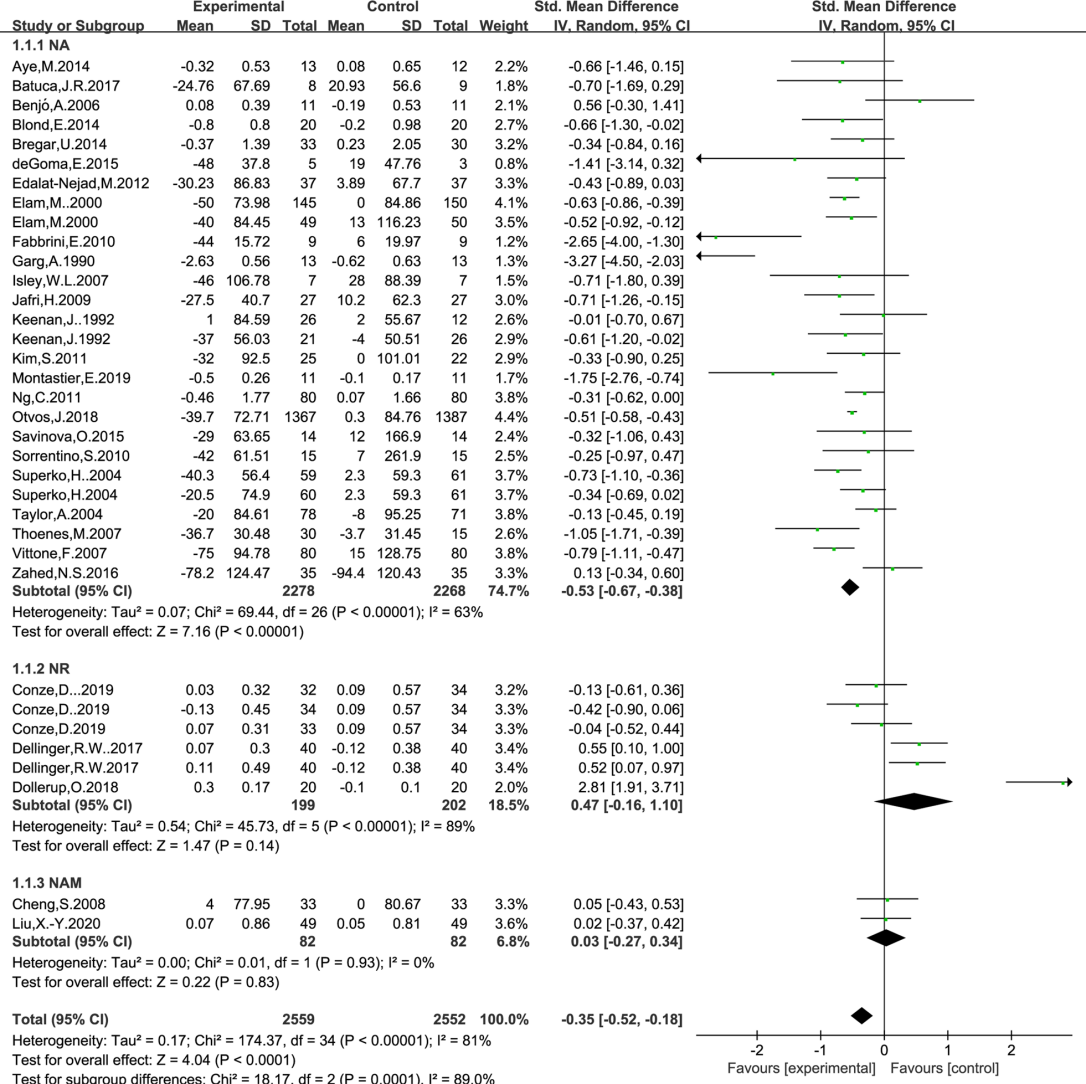
Effect of NAD+ precursor supplementation on TG

The data for determining the effect of NAD+ precursor supplementation on TG level was available in 29 trials (NA24, NR3, NAM2), including 2559 cases in the drug group and 2552 cases in the control group. The random-effects model was used for analyses. The results of meta-analysis showed that: NAD+ precursor can significantly reduce TG level in the patients (SMD = − 0.35, 95% CI (− 0.52, − 0.18), *P* < 0.0001; Fig. 3). Subgroup analysis showed that there was statistically significant difference in supplemental NA (SMD = − 0.53, 95% CI (− 0.67, − 0.38), *P* < 0.00001; Fig. 3), while there was no statistically significant difference in supplemental NR and NAM (*P* = 0.14 and *P* = 0.83; Fig. 3). No significant publication bias was found in the results of Begg’s plots (*P* = 1.80 and Egger’s test (*P* = 0.058) for TG.

### Effect of NAD+ precursor supplementation on TC level

**Fig. 4:**
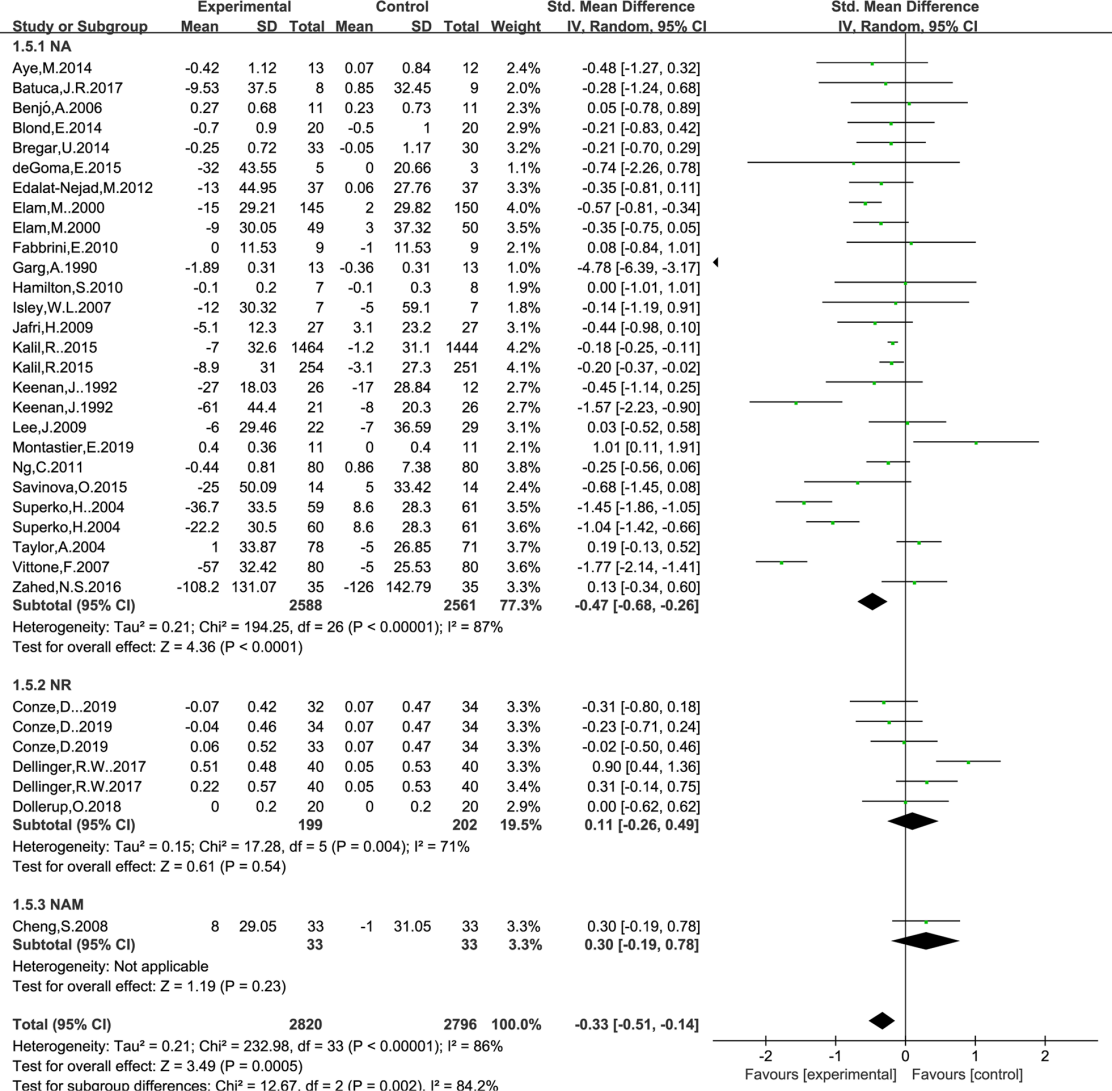
Effect of NAD+ precursor supplementation on TC

The data for determining the effect of NAD+ precursor supplementation on TC level was available in 27 trials (NA23, NR3, NAM1), including 2820 cases in the drug group and 2796 cases in the control group. The random-effects model was used for analyses. The results of meta-analysis showed that: NAD+ precursor can significantly reduce TC level in the patients (SMD = − 0.33, 95% CI (− 0.51, − 0.14), *P* = 0.0005; Fig. 4). Subgroup analysis showed that there was statistically significant difference in supplemental NA (SMD = − 0.47, 95% CI (− 0.68, − 0.26), *P* < 0.0001; Fig. 4), while there was no statistically significant difference in supplemental NR and NAM (*P* = 0.54 and *P* = 0.23). No significant publication bias was found in Begg’s plots (*P* = 1.54) and Egger’s test (*P* = 0.16) for TC.

### Effect of NAD+ precursor supplementation on LDL level

**Fig. 5:**
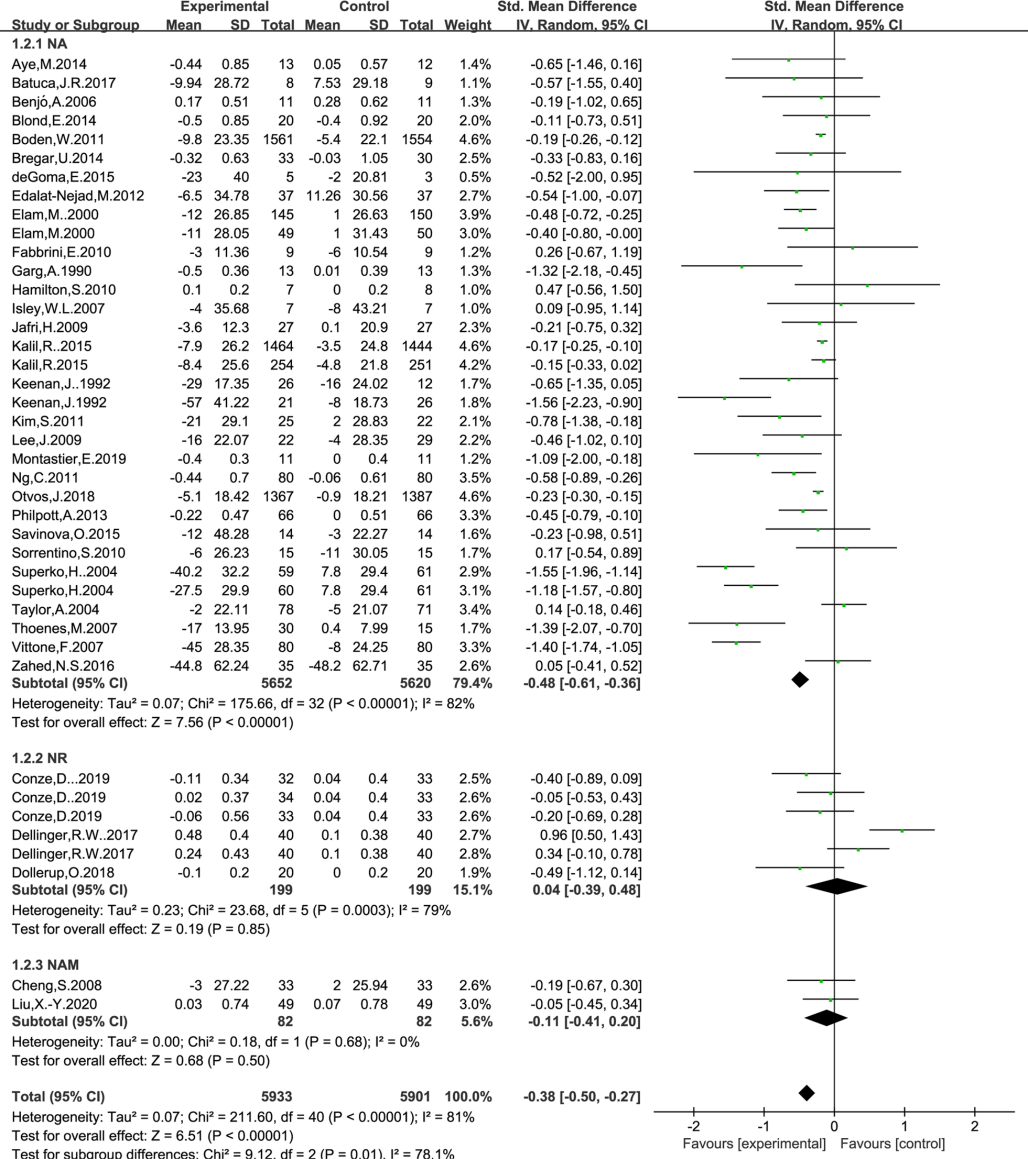
Effect of NAD+ precursor supplementation on LDL

The data for determining the effect of NAD+ precursor supplementation on LDL level were available in 34 trials (NA29, NR3, NAM2), including 5933 cases in the drug group and 5901 cases in the control group. The random-effects model was used for analyses. The results of meta-analysis showed that: NAD+ precursor can significantly reduce LDL level in the patients (SMD = − 0.38, 95% CI (− 0.50, − 0.27), *P* < 0.00001; Fig. 5). Subgroup analysis showed that there was statistically significant difference in supplemental NA (SMD = − 0.48, 95% CI (− 0.61, − 0.36), *P* < 0.00001; Fig. 5), while there was no statistically significant difference in supplemental NR and NAM (*P* = 0.85 and *P* = 0.50). NO significant publication bias was found in Begg’s plots (*P* = 1.51) and Egger’s test (*P* = 0.64) for LDL level.

### Effect of NAD+ precursor supplementation on HDL level

**Fig. 6:**
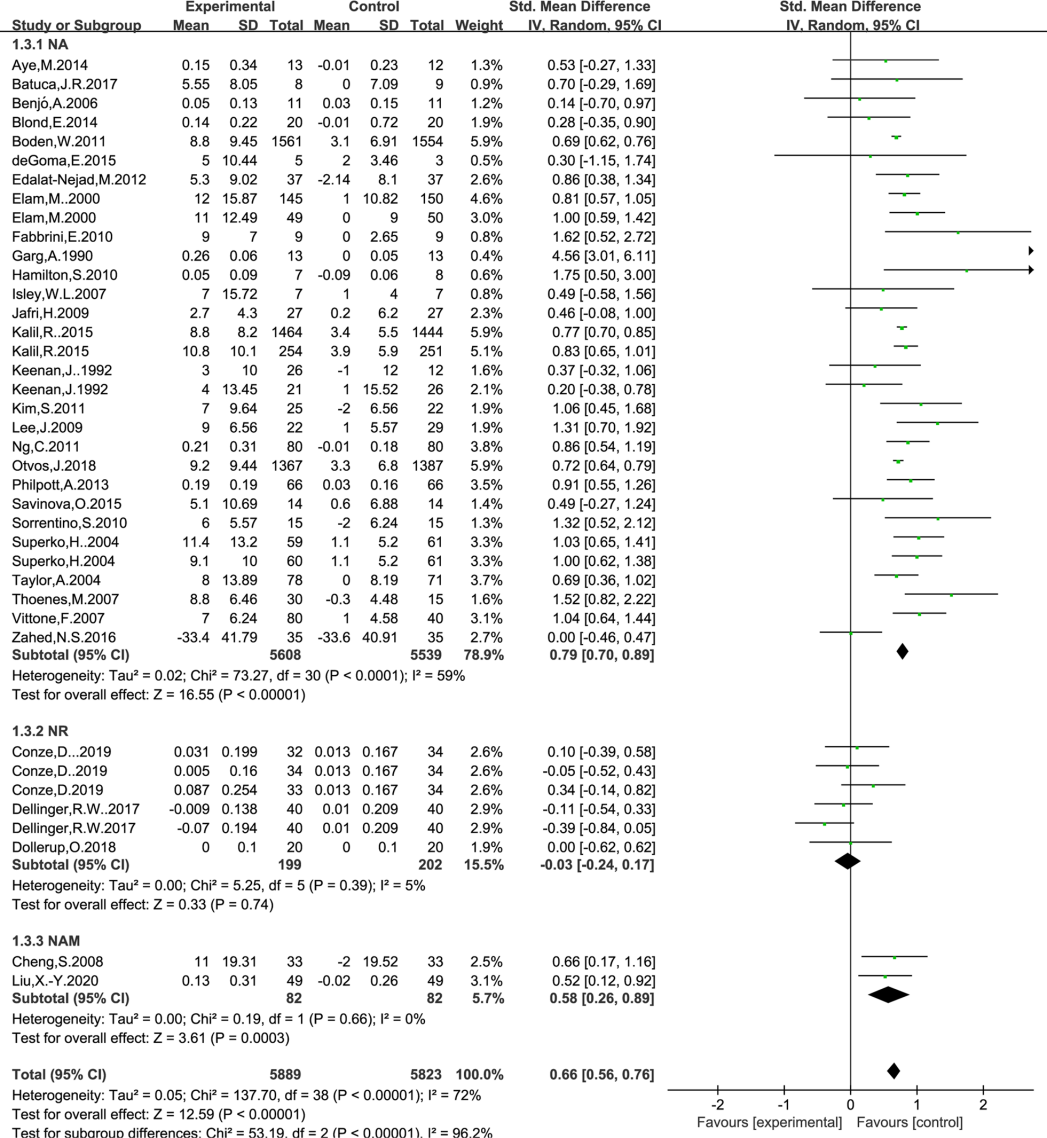
Effect of NAD+ precursor supplementation on HDL

The data for determining the effect of NAD+ precursor supplementation on plasma glucose was available in 32 trials (NA27, NR3, NAM2), including 5889 cases in the drug group and 5823 cases in the control group. The random-effects model was used for analyses. The results of meta-analysis showed that: NAD+ precursor can significantly increase HDL level in the patients (SMD = 0.66, 95% CI (0.56, 0.76), *P* < 0.00001; Fig. 6). Subgroup analysis showed that there was statistically significant difference in supplemental NA and NAM (SMD = 0.79, 95% CI (0.70, 0.89), *P* < 0.00001; Fig. 6) and (SMD = 0.58, 95% CI (0.26, 0.89), *P* = 0.0003; Fig. 6), while there was no statistically significant difference in supplemental NR (*P* = 0.74). No significant publication bias was found in Begg’s plots (*P* = 0.66) and Egger’s test (*P* = 0.073) for HDL.

### Effect of NAD+ precursor supplementation on fasting plasma glucose level

**Fig. 7:**
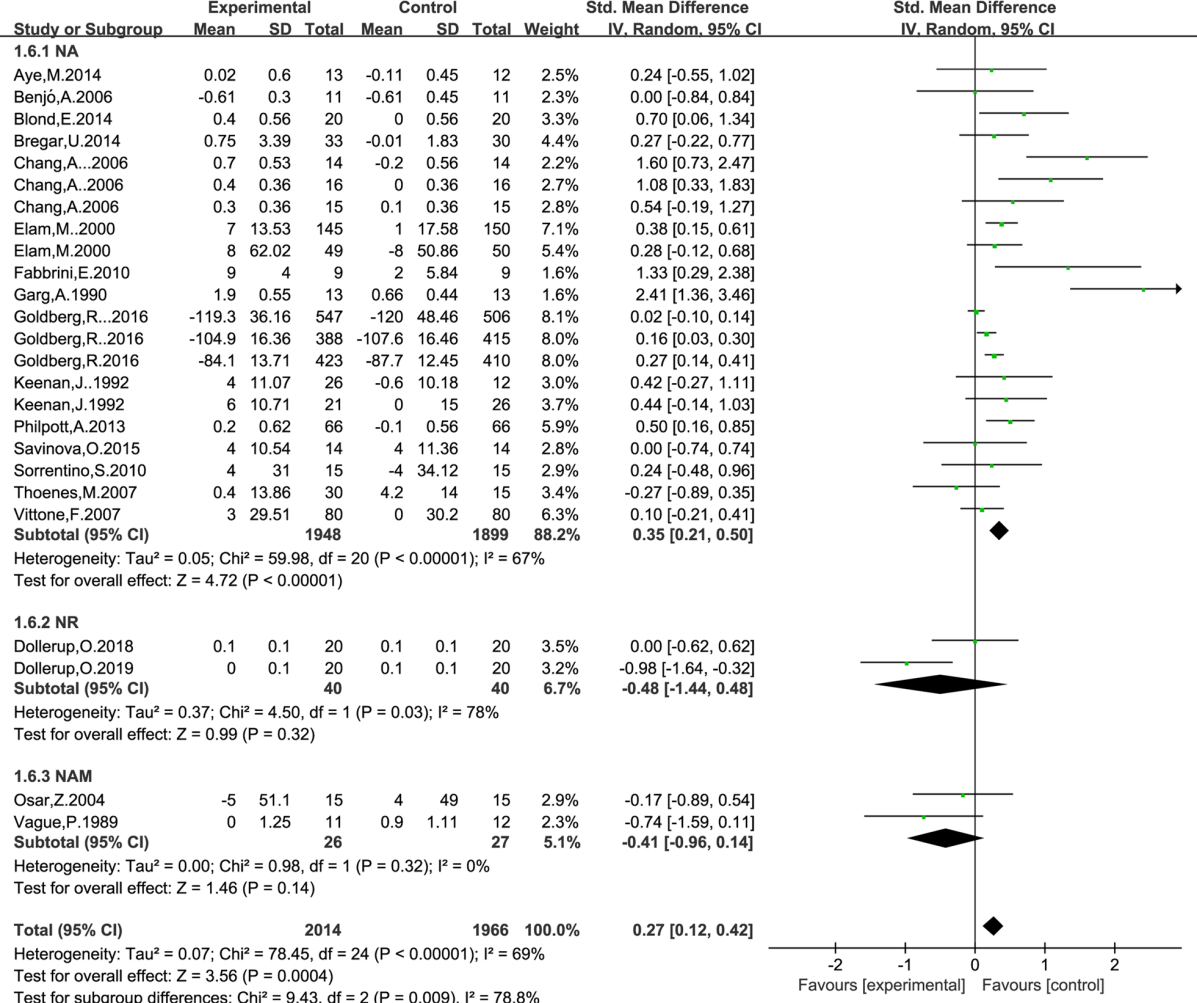
Effect of NAD+ precursor supplementation on Fasting plasma glucose

The data for determining the effect of NAD+ precursor supplementation on plasma glucose level was available in 19 trials (NA15, NR2, NAM2), including 2014 cases in the drug group and 1966 cases in the control group. The random-effects model was used for analyses. The results of meta-analysis showed that: NAD+ precursor can significantly increase plasma glucose level in the patients (SMD = 0.27, 95% CI (0.12, 0.42), *P* = 0.0004; Fig. 7). Subgroup analysis showed that there was statistically significant difference in supplemental NA (SMD = 0.35, 95% CI (0.21, 0.50), *P* < 0.00001; Fig. 6), while there was no statistically significant difference in supplemental NR and NAM (*P* = 0.32 and *P* = 0.14). No significant publication bias was found in Begg’s plots (*P* = 0.34) and Egger’s test (*P* = 0.18) for the plasma glucose level.

### According to the different health status of patients to Subgroup analysis

To make the study comprehensive, we included all the data we could collect in the study. Due to the different health status of patients, we divided all patients into 6 groups for subgroup analysis. The results are shown in Table 2. These six groups are (1) healthy people: We default to healthy people without special instructions in the article. (2) Dyslipidemia: including abnormal levels of HDL, LDL, TC and TG; (3) Pathoglycemia: including impaired glucose tolerance and diabetes mellitus; (4) Cardiovascular diseases: including atherosclerosis, coronary heart disease, old myocardial infarction, etc.; (5) Chronic kidney disease (CKD); (6) Others.

**Table 2:** Subgroup analysis

Subgroup	TG	TC	LDL	HDL	Fasting plasma glucose
Healthy people	SMD = 0.33, 95% CI = (− 0.23, 0.88), *P* = 0.25	SMD = 0.00, 95% CI = (− 0.37, 0.38), *P* = 0.99	SMD = − 0.06, 95% CI = (− 0.43, 0.32), *P* = 0.76	SMD = 0.12, 95% CI = (− 0.24, 0.48), *P* = 0.51	SMD = 0.33, 95% CI = (0.16, 0.50), *P* = 0.0001
CKD	SMD = − 0.05, 95% CI = (− 0.30, 0.19), *P* = 0.66	SMD = − 0.08, 95% CI = (− 0.33, 0.18), *P* = 0.56	SMD = − 0.16, 95% CI = (− 0.30,− 0.02), *P* = 0.02	SMD = 0.60, 95% CI = (0.31, 0.89), *P* < 0.0001	-
Dyslipidemia	SMD = − 0.47, 95% CI = (− 0.63,− 0.31), *P* < 0.00001	SMD = − 0.68, 95% CI = (− 1.07 ,− 0.29), *P* = 0.0006	SMD = − 0.80, 95% CI = (− 1.18, − 0.41), *P* < 0.0001	SMD = 0.79, 95% CI = (0.63, 0.96), *P* < 0.00001	SMD = 0.27, 95% CI = (− 0.08, 0.61), *P* = 0.13
Pathoglycemia	SMD = − 1.83, 95% CI = (− 4.52, 0.86), *P* = 0.18	SMD = − 1.56, 95% CI = (− 3.61,− 0.49), *P* = 0.14	SMD = − 0.45, 95% CI = (− 1.26, 0.36), *P* = 0.27	SMD = 2.32, 95% CI = (0.44, 4.20), *P* = 0.02	SMD = 0.31, 95% CI = (− 0.01, 0.63), *P* = 0.06
Cardiovascular disease	SMD = − 0.52, 95% CI = (− 0.60,− 0.45), *P* < 0.00001	SMD = − 0.69, 95% CI = (− 1.51, 0.13), *P* = 0.10	SMD = − 0.48, 95% CI = (− 0.88,− 0.08), *P* = 0.02	SMD = 0.73, 95% CI = (0.66, 0.80), *P* = < 0.00001	SMD = 0.28, 95% CI = (0.03, 0.54), *P* = 0.03
Other	SMD = − 0.92, 95% CI = (− 1.68,− 0.16), *P* = 0.02	SMD = 0.14, 95% CI = (− 0.20, 0.49), *P* = 0.42	SMD = − 0.18, 95% CI = (− 0.43, 0.07), *P* = 0.15	SMD = 0.84, 95% CI = (0.59, 1.09), *P* = < 0.00001	SMD = 0.15, 95% CI = (− 0.74, 1.04), *P* = 0.74

-: there is only one sample or no sample in the subgroup

It can be found that the supplement of NAD+ precursors seems to have little effect on healthy people, but it has a significant beneficial effect on patients with cardiovascular disease and dyslipidemia.

## Discussion

In this study, a comprehensive meta-analysis of data from currently published clinical trials with NAD+ precursors showed that supplementation with NAD+ precursors improved the levels of TG, TC, LDL and HDL in humans compared with placebo or no treatment but resulted in high glucose levels. Among them, NA has the most significant effect on improving lipid metabolism. Currently, there is no meta-analysis on the effect of NAD+ precursors on lipid metabolism in the human body. Ding et al., performed a meta-analysis of 7 randomized controlled trials showing that NA alone or in combination significantly improved dyslipidemia in patients with T2DM, but glucose levels need to be monitored during long-term treatment [12]. Xiang et al., conducted a meta-analysis of 8 randomized controlled trials and found that NA supplementation can improve the level of blood lipid without affecting the level of blood glucose in patients with type 2 diabetic mellitus (T2DM) [13]. However, these studies were limited to patients with T2DM. This study was based on existing clinical RCTs to evaluate the effect of NAD+ supplementation on lipid control in humans. The comprehensive results showed that supplementation with NAD+ precursors significantly improved lipid metabolism in humans.

NA is widely used to regulate dyslipidemia and treat atherosclerotic cardiovascular disease. Studies have shown that niacin can reduce plasma TC, TG, and LDL levels, and increase HDL level. In addition, various clinical trials have shown that niacin therapy significantly reduces overall mortality from various cardiovascular diseases and delays the progression of atherosclerosis [14, 15]. Jin et al., used a human hepatoblastoma cell line (HepG2) model to study the relationship between NA and intracellular ApoB, and the results showed that NA significantly increased the degradation of intracellular ApoB [16]. NA inhibits the synthesis of TG through a variety of mechanisms, which may hinder the lipidation and transport of ApoB on the endoplasmic omentum, and may create a favorable environment for intracellular ApoB degradation. ApoB is the major protein of very low-density lipoproteins, intermediate-density lipoproteins, LDL and lipoprotein (a). These ApoB-containing lipoproteins (especially elevated LDL levels) are associated with accelerated atherosclerosis, and their decrease can delay the progression of atherosclerosis. It has been found that oral administration of 200 mg of NA daily can reduce plasma free fatty acid (FFA) concentration [17]. NA may inhibit the mobilization of adipose tissue by inhibiting the activity of triacylglycerase in adipose tissue, and reduce the release of free fatty acids in adipose cells, leading to a decrease in plasma FFA concentration and liver uptake of FFA, resulting in a decrease in TG synthesis and LDL secretion. Diacylglycerol acyltransferase (DGAT2) is the key enzyme in TG synthesis, and NA can directly inhibit the activity of liver DGAT2, but has no effect on DGAT1 [18]. Hu et al., treated 39 patients with dyslipidemia with 2 g of ERN per day, and the results showed that plasma TG and liver fat contents decreased significantly, which was speculated to be caused by NA inhibiting hepatic DGAT2 [19]. However, it is worth noting that NA can lower blood lipids and is used to treat dyslipidemia, but at doses greater than 50 mg/day, NA can also cause flushing [20].

Nicotinamide riboside is a vitamin that occurs naturally in the human diet and is one of the precursors of NAD+ . In animal models, NR supplementation can improve glucose tolerance and reduce metabolic abnormalities in mice [21]. The study from Conze et al., showed that NR supplementation can improve the level of human lipid metabolism [1], which plays a role by activating sirtuins to regulate human metabolism. In rodents, NR is more efficient in boosting NAD+ than NA and NAM [22], but the number of clinical studies on NR intervention is relatively low, and the evidence of NR's benefit to human beings is limited.

Both NAM and NA are the main forms of vitamin B3 and, despite their similar structure, do not have the same effects. Currently, NAM is a commonly used treatment of dialysis patients with renal insufficiency to improve hyperphosphatemia in clinical. In previous reports, nicotinamide has no significant effect on human lipid metabolism [7], but in recent years, more and more studies have shown that nicotinamide can significantly improve the level of lipid metabolism in patients. Liu, X.Y. et al. studied 98 hemodialysis patients treated with NAM 500–1500 mg daily, and the results showed that after 52 weeks of intervention, the blood lipid level of the patients was significantly improved compared with placebo, and the blood glucose was not increased [9]. Cheng, et al. treated 33 patients with long-term hemodialysis with NAM 500–1500 mg daily, and after 8 weeks, the blood phosphorus level of the patients decreased significantly and the blood lipid level improved significantly [10]. The study of Takahashi et al., also showed that NAM treatment could improve patients' lipid metabolism [11]. At present, the number of studies on the improvement of human lipid metabolism by nicotinamide intervention is limited, and the mechanism remains unclear, but the existing studies have shown its great clinical value.

We divided all patients into 6 groups for subgroup analysis. It was found that the supplement of NAD+ precursors seems to have little effect on healthy people, but it has a significant beneficial effect on patients with cardiovascular disease and dyslipidemia. Limitations of this study include the variation in study design, in the selection of inclusion articles, and reporting of the biochemical parameter. (1) In terms of study design, there were varying doses of the study medication, and some of the trials enforced strict diet and exercise regimens in addition to NAD+ precursor supplementation, or took simvastatin and Ezetimibe to background treatment, while others only supplemented NAD and did not incorporate any lifestyle modification into the design. In addition, inclusion criteria varied with some trials allowing diabetics, while others excluding such patients. (2) This study only includes English literature, which may affect the inference of results; The sample size included in the study varies greatly, which may lead to some heterogeneity. (3) In the report of biochemical parameters, some studies use mg/dl as the unit and some use μmol/L, which makes it difficult to collect data. Due to the limited number of published studies, the heterogeneity of efficacy of different precursors is greatly affected by study samples, and needs to be verified by more high-quality studies.

## Conclusion

In this study, a meta-analysis based on currently published clinical trials with NAD+ precursors showed that supplementation with NAD+ precursors improved TG, TC, LDL, and HDL levels in humans, but resulted in hyperglycemia, compared with placebo or no treatment. Among them, NA has the most significant effect on improving lipid metabolism. In addition, although NR and NAM supplementation had no significant effect on improving human lipid metabolism, the role of NR and NAM could not be directly denied due to the few relevant studies at present. Based on subgroup analysis, we found that the supplement of NAD+ precursors seems to have little effect on healthy people, but it has a significant beneficial effect on patients with cardiovascular disease and dyslipidemia. Due to the limitation of the number and quality of included studies, the above conclusions need to be verified by more high-quality studies.

## Supplementary Information

**Additional file 1.** Search strategy for the meta-analysis.

## Data Availability

The datasets used and/or analysed during the current study available from the corresponding author on reasonable request.

## Abbreviations

CIs: Confidence intervals
DGAT2: Diacylglycerol acyltransferase
HDL: High-density lipoprotein
LDL: Low-density lipoprotein
NA: Nicotinic Acid
NAM: Nicotinamide
NMN: Nicotinamide Mononucleotide
NR: Nicotinamide Riboside
QA: Quinolinic acid
RCT: Randomized controlled trial
SMD: Standardized Mean Difference
T2DM: Type 2 diabetic mellitus
TC: Total cholesterol
TG: Triglyceride
