# Supplementary material for: Effects of NAD+ precursor supplementation on glucose and lipid metabolism in humans: a meta-analysis
Source: Nutr Metab (Lond). 2022 Mar 18;19:20. doi: 10.1186/s12986-022-00653-9 (PMC8932245; doi:10.1186/s12986-022-00653-9)
Supplement: Supplementary file 1 — Additional file 1. Search strategy for the meta-analysis. [file 12986_2022_653_MOESM1_ESM.docx]

**Additional file 1**

**Appendix 1.Pubmed search strategy**

Searched February 5, 2021

((((("NAD"[MeSH Terms] OR "NAD precursor"[Title/Abstract] OR "Nicotinic Acids"[MeSH Terms] OR "Nicotinic Acid"[Title/Abstract] OR "NA"[Title/Abstract] OR "Niacin"[MeSH Terms] OR "Niacin"[Title/Abstract] OR "Niacinamide"[MeSH Terms] OR "Niacinamide"[Title/Abstract] OR "Nicotinamide"[Title/Abstract] OR "NAM"[Title/Abstract] OR "Nicotinamide Riboside"[Title/Abstract] OR "NR"[Title/Abstract] OR "Nicotinamide Mononucleotide"[MeSH Terms] OR "Nicotinamide Mononucleotide"[Title/Abstract] OR "NMN"[Title/Abstract]) AND ("Randomized Controlled Trial"[Publication Type] OR "Controlled Clinical Trial"[Publication Type] OR "Clinical Trials as Topic"[MeSH Terms:noexp] OR "randomized"[Title/Abstract] OR "placebo"[Title/Abstract] OR "randomly"[Title/Abstract] OR "trial"[Title/Abstract])) NOT "Animals"[MeSH Terms]) NOT "mice"[Title/Abstract]) NOT "mouse"[Title/Abstract]) NOT "review"[Title/Abstract] (1,248)

**Appendix 2.Embase search strategy**

Searched February 5, 2021

#1 'nad'/exp OR 'nad precursor':ab,ti OR 'nicotinic acids'/exp OR 'nicotinic acids':ab,ti OR 'na':ab,ti OR 'niacin'/exp OR 'niacin':ab,ti OR 'niacinamide'/exp OR 'niacinamide':ab,ti OR 'nicotinamide':ab,ti OR 'nam':ab,ti OR 'nicotinamide riboside'/exp OR 'nicotinamide riboside':ab,ti OR 'nr':ab,ti OR 'nicotinamide mononucleotide'/exp OR 'nicotinamide mononucleotide':ab,ti OR 'nmn':ab,ti (316,157)

#2 ('randomized controlled trial'/exp OR 'controlled clinical trial'/exp OR 'clinical trials as topic'/exp OR 'randomized':ab,ti OR 'placebo':ab,ti OR 'randomly':ab,ti OR 'trial':ab,ti) NOT 'animals'/exp NOT 'mice':ab,ti NOT 'mouse':ab,ti NOT 'review':ab,ti (135,543)

#3 #1 AND #2 (1,088)

**Appendix 3.The CENTRAL (Cochrane Central Registry of Controlled Trials) search strategy**

Searched February 6, 2021

#1 MeSH descriptor:[NAD] explode all trees(37)

#2 (NAD precursor):ti,ab,kw(46)

#3 MeSH descriptor:[Nicotinic Acid] in all MeSH products (2221)

#4 (Nicotinic Acid):ti,ab,kw(681)

#5 MeSH descriptor:[Niacin] in all MeSH products (591)

#6 (Niacin):ti,ab,kw(1131)

#7 MeSH descriptor:[Niacinamide] explode all trees (1150)

#8 (Niacinamide):ti,ab,kw(980)

#9 (Nicotinamide):ti,ab,kw(791)

#10 (NAM):ti,ab,kw(666)

#11 (Nicotinamide Riboside):ti,ab,kw(53)

#12 (NR):ti,ab,kw(1588)

#13 MeSH descriptor:[ Nicotinamide Mononucleotide] explode all trees (0)

#14 (Nicotinamide Mononucleotide):ti,ab,kw(21)

#15 (NMN):ti,ab,kw(23)

#16 #1 OR #2 OR #3 OR #4 OR #5 OR #6 OR #7 OR #8 OR #9 OR #10 OR #11 OR #12 (4512)

**Appendix 4.Web of Science search strategy**

Searched February 6, 2021

#1 TS=(NAD OR Nicotinic Acids OR Niacin OR Niacinamide OR Nicotinamide Riboside OR Nicotinamide Mononucleotide) OR AB=(NAD precursor OR Nicotinic Acids OR NA OR Niacin OR Niacinamide OR Nicotinamide OR NAM OR Nicotinamide Riboside OR NR OR Nicotinamide Mononucleotide OR NMN) (108,580)

#2 (TS=(Randomized Controlled Trial OR Controlled Clinical Trial OR Clinical Trials as Topic) OR AB=(randomized OR placebo OR randomly OR trial)) NOT TS=(Animals) NOT AB=(mice) NOT AB=(mouse) NOT AB=(review) (488,520)

#3 #1 AND #2 (3,097)

**Appendix 5.Scopus search strategy**

Searched February 6, 2021

( TITLE-ABS ( nad ) OR TITLE-ABS ( "NAD precursor" ) OR TITLE-ABS ( "Nicotinic Acids" ) OR TITLE-ABS ( na ) OR TITLE-ABS ( niacin ) OR TITLE-ABS ( niacinamide ) OR TITLE-ABS ( nicotinamide ) OR TITLE-ABS ( nam ) OR TITLE-ABS ( "Nicotinamide Riboside" ) OR TITLE-ABS ( nr ) OR TITLE-ABS ( "Nicotinamide Mononucleotide" ) OR TITLE-ABS ( nmn ) ) AND ( ( TITLE-ABS ( randomized ) OR TITLE-ABS ( placebo ) OR TITLE-ABS ( randomly ) OR TITLE-ABS ( trial ) ) not TITLE-ABS ( animals ) OR TITLE-ABS ( mice ) OR TITLE-ABS ( mouse ) OR TITLE-ABS ( review ) ) (1,991)
